# A cross-lagged network analysis of multisystemic factors influencing adolescent depressive symptoms: considering gender differences

**DOI:** 10.1186/s13034-025-00945-x

**Published:** 2025-07-30

**Authors:** Qiu Li, Xue-Ying Liu, Wei-Liang Wang, Fang Zhou

**Affiliations:** School of Nursing, Xuzhou Medical University, Xuzhou, China

**Keywords:** Depression, Resilience, Adolescent, Network analysis

## Abstract

**Background:**

Adolescent depression has become a pressing public health concern in China, with recent estimates indicating a prevalence rate of 26.17%. This mental health issue poses significant risks to adolescents’ psychological development and long-term well-being. The present study investigates how multiple resilience-related factors across emotional, familial, school, and social domains interact with depressive symptoms over time, with particular attention to gender differences.

**Methods:**

A cross-lagged network analysis was conducted using longitudinal data from 770 adolescents recruited from two middle schools in northern China. Participants completed various validated questionnaires measuring depression, emotional resilience, family resilience, teacher support, friendship quality, and social support at two time points.

**Results:**

Network analysis revealed that teacher support had the highest out-expected influence for the overall sample, while friend support was central to in-expected influence. Gender differences were pronounced; male adolescents primarily relied on friendship quality, whereas female adolescents benefitted more from teacher support. Additionally, depression had a greater weakening effect on social support in males and on family support in females.

**Discussions:**

This study highlights gender-specific pathways in the interaction between depressive symptoms and resilience factors among adolescents. The findings suggest that both teacher and peer support are critical in shaping these dynamics, with implications for developing targeted interventions aimed at enhancing emotional resilience and addressing depressive symptoms in a gender-sensitive manner.

## Introduction

With the advancement of society and the intensification of competitive pressure, mental health issues among adolescents have become increasingly prominent. In China, the overall prevalence of depressive symptoms among adolescents has reached 26.17% [1, 2]. Depression in adolescents not only affects their current psychological well-being but may also lead to long-term mental health issues, such as various behavioral problems and an increased risk of suicide, thereby contributing to a greater disease burden [3]. Early intervention for depressive symptoms is crucial for health throughout the entire lifespan.

However, depressive symptoms do not develop in isolation, they are embedded within a broader psychosocial context and influenced by multiple external factors across individual and environmental domains. Bronfenbrenner’s ecological systems theory [4] provides a valuable framework for conceptualizing these influences, emphasizing that adolescent development occurs within multiple, nested systems. Precisely because of the complexity of adolescent mental health issues, the social-ecological model is often utilized to explain the multidimensional characteristics of depressive symptoms. The social-ecological model reveals that individual health is dynamically influenced by personal traits, psychological factors, close relationships, community environments, and macro-level social structures [5]. Existing research has indicated that individual factors include personality traits, knowledge, skills, and cognitive social capital [6, 7]. The micro-level refers to daily interactions with family, peers, and schools, which directly shape values and behavioral patterns—quality social support, healthy peer relationships, strong family bonds, positive relationships, and enriching school experiences can enhance mental health [8–13].

Previous studies have shown that, the microsystem—including family, school, peer groups, and social community—exerts the most proximal and significant impact. Emotion regulation ability, perceived family support, teacher emotional support, peer relationships, and broader social support structures all constitute key elements of this microsystem and have been shown to play important roles in adolescent mental health [14–17]. Ungar et al. [18] also emphasizes in his research that resilience results from the interaction between an individual’s own capabilities and multiple system resources, and focusing solely on symptom suppression at the individual level is unlikely to yield effective outcomes. Supportive interactions within and across these systems can buffer against stress and reduce the risk of depressive symptoms, while dysfunctional dynamics may exacerbate vulnerability. There is an urgent need to identify and understand the drivers of adolescent depression to provide intervention targets for prevention.

However, most existing studies have focused on isolated domains—such as parenting, peer relationships, or emotional functioning—without integrating these factors within a broader ecological context [19–23]. In recent years, the application of psychological network analysis has seen considerable use in the interpretation of multivariate relationships. Compared to traditional latent variable analyses, such as structural equation modeling, assessing complex multivariate relationships often requires pre-establishing causal paths and selecting predictor, mediator, and outcome variables. Given the complexity of adolescent depression-related factors and the potential for bidirectional relationships, these prerequisites do not always hold [24]. Network analysis offers a data-driven alternative [25, 26]: it does not rely on a priori causal models but generates spatialized networks that position the most central variables at the core, thereby intuitively presenting the interactions and reinforcements both within the same construct and across different constructs. Additionally, the cross-sectional nature of much of this research limits inferences about directionality and causality. Cross-lagged panel network analysis (CLPN) addresses these limitations by incorporating longitudinal data to investigate temporal dynamics and bidirectional effects between depressive symptoms and contextual variables [22, 23, 27, 28].

Research on the network mechanisms of depressive symptoms in adolescents increasingly reveals structural gender differences in the pathological processes. Studies have found that female symptom networks typically exhibit higher connectivity density, with anxiety (e.g., “excessive worry”) serving as a core node, while male networks are more centered around depression (e.g., “low mood”) as a hub [29]. During puberty, increased estrogen enhances 5-HT function and strengthens amygdala–prefrontal connectivity, making girls’ emotional nodes more tightly linked [30]. They also exhibit a more prolonged HPA-axis response to stress, which promotes the “sadness–self-blame” cycle [31, 32]. Meanwhile, girls often use rumination and internal attributions to reinforce emotional coupling, whereas boys lean toward external attributions and problem-solving, reducing such connections [33]. On a social level, girls are more sensitive to comparisons with close peers, and relationship setbacks rapidly trigger a “social withdrawal–self-blame” cluster that spreads within their network; boys, by contrast, tend to express conflict through overt behaviors, resulting in weaker emotional network feedback [34].In a studies from a social ecology perspective, female symptom networks may form stronger feedback loops with factors such as domestic violence and self-loathing, while male networks are more influenced by factors such as academic pressure and impulsivity [35]. In addition, there are significant gender differences in stress perception, stress coping, and environmental sensitivity [36, 37]. Overall, gender differences are not only reflected in the phenotypic level of depression but are also likely to profoundly influence the dynamic evolution of symptom networks. However, little is currently known about the sex differences in external network mechanisms related to factors influencing depressive symptoms. This study aims to examine the impact mechanisms of resilience factors on depressive symptoms from an ecological perspective. By integrating dimensions of individual emotional resilience, family resilience, teacher emotional connections, friendship quality, and social support, we will use cross-lagged network analysis to validate how these resilience networks influence depressive symptoms.

## Methods

### Participants and procedures

This study included two middle schools located in a northern city in China using convenience sampling. Data were collected at two time points: October 2023 and November 2024. The study was approved by the Ethics Committee of Xuzhou Medical University (XZHMU-2023029). Informed consent was obtained from both the participants and their legal guardians prior to participation. After consent was secured, paper-based questionnaires were administered on-site. Participants completed the questionnaires during school hours and returned them immediately upon completion. All original data is securely maintained by the research team and monitored by the ethics committee. As feedback, the research results will be used for the development of subsequent intervention programs, contributing to adolescent mental health. The analysis report of the overall data is only shared with the psychology teachers at the target middle school to guide the school’s psychological education work.

### Measures

#### Demographics

A self-developed questionnaire was used, aligned with the objectives of the study, to collect demographic information, including age and gender.

#### Depression

The Children’s Depression Inventory (CDI) [38] was employed to measure depression. The CDI consists of 27 self-report items. Each item on the scale is scored as 0, 1, or 2, with higher scores indicating more severe depressive symptoms. A total score greater than 19 is considered indicative of depression. The reliability and validity of the Chinese version have been validated in several studies [39], with a Cronbach’s *α* coefficient of 0.86.

#### Individual emotional resilience

The Adolescents’ Emotional Resilience Questionnaire (AERQ) consists of two dimensions: the ability to generate positive emotions and the ability to recover from negative emotions, comprising a total of 11 items. Each item is rated on a 6-point scale, ranging from 1 (completely disagree) to 6 (completely agree). Higher total scores indicate greater emotional resilience, making the AERQ an effective measure of emotional resilience in adolescents [40]. In this study, the Cronbach’s *α* coefficient was 0.81.

#### Family resilience

Family resilience was measured using the Chinese version of the Family Resilience Rating Scale (FARS) [41]. The scale consists of 32 items that assess family resilience from three dimensions: family communication and problem-solving, utilization of socioeconomic resources, and family belief systems. The response options range from “strongly disagree” to “strongly agree,” with a scoring scale of 1 to 4, where higher scores indicate a higher level of family resilience. In this study, the Cronbach’s *α* coefficient was 0.96.

#### Teacher support

The Perceived Teachers’ Emotional Support Questionnaire (PTESQ) was utilized to assess the perceived emotional support provided by teachers [42]. The questionnaire consists of 18 items designed to measure four dimensions of perceived teacher emotional experiences: understanding, caring, respect, and encouragement. The items are rated on a five-point Likert scale, with higher scores indicating a greater level of understanding of the emotional support provided by teachers. In this study, the Cronbach’s *α* coefficient was 0.96.

#### Friend support

The revised Friendship Quality Questionnaire (FQQ) was employed to assess the quality of friendship between adolescents and their best friends [43, 44]. This questionnaire comprises six dimensions: affirmation and caring, help and support, companionship and recreation, intimacy and communication, conflict resolution strategies, and conflict and betrayal, totaling 18 items. Responses are rated on a five-point scale, where 1 indicates “strongly disagree” and 5 indicates “strongly agree.” In this study, the Cronbach’s *α* coefficient was 0.89.

#### Social support

The Social Support Rating Scale for Children and Adolescents (SSRS-CA) is a culturally adapted assessment tool developed based on the Social Support Rating Scale, specifically designed for this population [45]. It comprises three dimensions: objective support, subjective support, and the utilization of social support, totaling 10 items. In this study, the Cronbach’s *α* coefficient was 0.75.

### Data analysis

Statistical analyses were conducted using R version 4.40. Continuous variables were described as means and standard deviations, while categorical variables were presented as counts and percentages. The analysis compared the scores of male and female using independent samples t-tests to identify significant differences across variables. All *p* values were 2-tailed, and 0.05 was considered to indicate statistical significance.

The CLPN was estimated for the overall sample as well as separately for female and male subgroups using a series of regularized regression models to compute both autoregressive and cross-lagged effects across two time points [46]. For the overall model, both age and gender were included as covariates; for the gender-specific models, only age was statistically controlled. Autoregressive effects captured the coefficient of a symptom at T1 predicting itself at T2, controlling for all other symptoms at T1 and covariates. Cross-lagged effects involved the coefficient of a symptom at T1 predicting a different symptom at T2, after accounting for all other symptoms at T1 and covariates. The estimation process utilized penalized maximum likelihood with a lasso penalty on the regression coefficients [46]. This approach effectively reduced overfitting and eliminated trivial paths by shrinking small regression paths to zero, enhancing the generalizability of results [47]. The *glmnet* package [46] was employed for computing regularized regressions and the *qgraph* package [48] was used for visualization of the network. Based on previous research [49], we used the random forest imputation method provided by the *missForest* package [50], which created a single, imputed dataset for analysis, to deal with missing data. As the CLPN is a directed network derived from longitudinal data, node-level centrality was assessed using two indices: out-expected influence (out-EI) and in-expected influence (in-EI). Out-EI reflects the degree to which a node at T1 predicts other nodes at T2 (sum of outgoing edge weights), whereas in-EI quantifies how strongly a node at T2 is predicted by other nodes at T1 (sum of incoming edge weights). A higher in-EI value indicates that the node is more predictable by other nodes in the network. A higher out-EI value indicates that the node has a stronger ability to predict other nodes. Together, these measures offer insight into each variable’s temporal influence and vulnerability within the network [51].

Subsequently, the accuracy and stability of the network were assessed using the *bootnet* package [52]. To evaluate the stability of the network, case-drop bootstrapping was used to estimate the stability of rank-order centrality indices and calculate correlation stability (CS) coefficients. A CS coefficient greater than 0.25 was considered acceptable, while a coefficient greater than 0.50 was considered excellent [52].

## Results

A total of 770 valid samples were included in this study, the mean age of the overall sample at baseline was 16.06 years (SD = 1.44). Among the participants, 495 were female, accounting for 64.28% of the overall sample. Overall, in terms of resilience, male and female scores differ across various dimensions, as shown in Table 1.

**Table 1:** Characteristics and resilience scores among the overall sample and subgroups. Characteristics of the overall sample, overall male sample, overall female sample, along with a comparison of depression and psychological resilience

	Overall Sample *n*= (770) Female= (64.28%)	Male Sample *n*= (275)	Female Sample *n*= (495)	*P* value
Age, Year (T1)	16.06(1.44)	15.90(1.55)	16.20(1.36)	0.0075
CDI (T1)	17.73(6.94)	17.93(7.02)	17.61(6.91)	0.4740
CDI (T2)	14.80(8.20)	17.82(7.60)	13.13(8.06)	0.0000
AERQ (T1)	34.51(10.07)	36.72(10.20)	33.29(9.80)	0.0000
AERQ (T2)	36.11(9.05)	36.92(9.06)	35.66(9.02)	0.0934
FARS (T1)	91.33(17.67)	90.14(21.19)	91.99(15.34)	0.4721
FARS (T2)	94.18(14.67)	94.99(15.68)	93.73(14.06)	0.1355
FQQ (T1)	63.87(16.15)	60.72(18.41)	65.62(14.47)	0.0005
FQQ (T2)	65.45(16.86)	58.37(16.79)	69.38(15.58)	0.0000
PTESQ (T1)	58.47(17.42)	56.87(19.76)	59.36(15.92)	0.0180
PTESQ (T2)	57.28(13.67)	57.49(15.80)	57.16(12.34)	0.4679
SSRS-CA (T1)	28.52(9.93)	27.55(11.29)	29.60(9.05)	0.0405
SSRS-CA (T2)	29.40(13.50)	28.38(14.65)	29.96(12.81)	0.0352

*CDI* children’s depression inventory, *AREQ* the adolescents’ emotional resilience questionnaire, *FARS* the family resilience rating scale, *FQQ* friendship quality questionnaire, *PTESQ* perceived teachers' emotional support questionnaire, *SSRS-CA* social support rating scale for children and adolescents

### Network in the overall sample

**Fig. 1:**
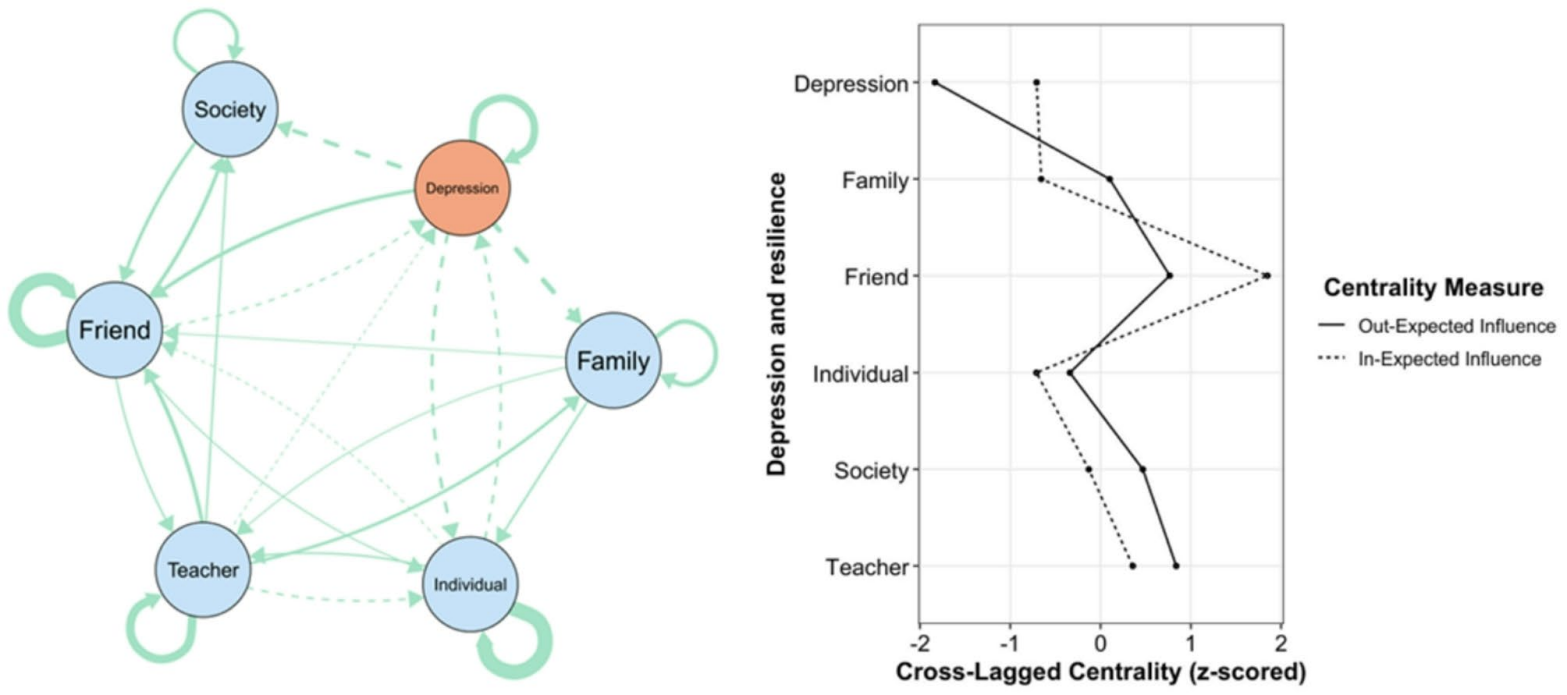
Overall sample cross-lag network and Standardized centrality estimates of depressive symptoms network

The network and standardized centrality estimates are presented in Fig. 1. Teacher exhibited the highest out-EI (0.16). Friend had the highest in-EI (0.36). The strongest positive associations were Teacher → Friend (*β* = 0.12). The strongest negative associations were Depression → Family (*β* = −0.14).

### Gender-Specific networks

A notable finding is that the edge lists between the two networks were not significantly correlated (*r* = 0.30, *p* = 0.09), with almost half of the edges (53.33%) having different directions. This indicates potential differences in node connections between the two networks. Regarding central symptoms, the person correlation of the overall out-EI between the two networks was 0.74, though it was not statistically significant (*p* = 0.092). Similarly, the correlation for the overall in-EI was weaker (*r* = 0.22, *p* = 0.66), suggesting differences in central depressive symptoms between genders.

**Fig. 2:**
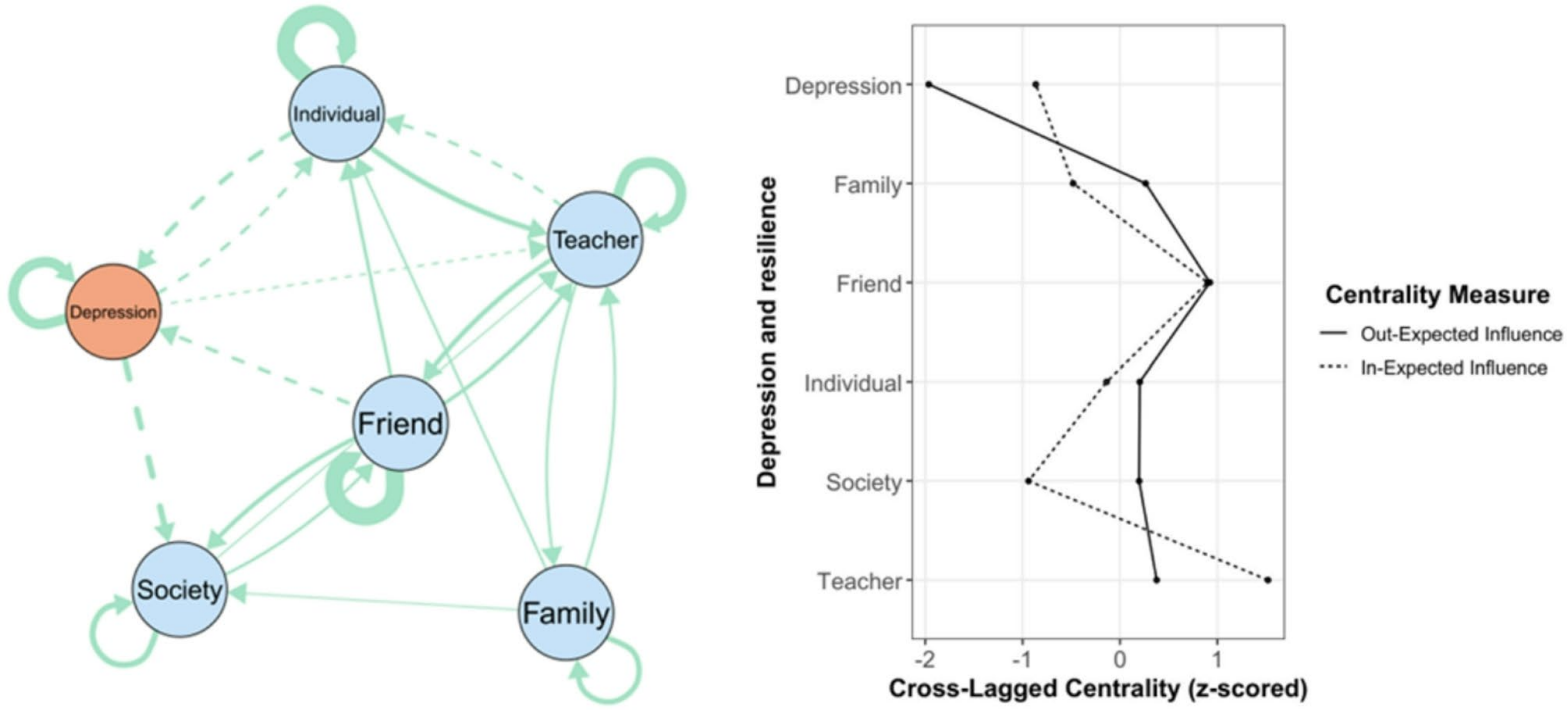
Male sample cross-lag network and Standardized centrality estimates of depressive symptoms network

**Fig. 3:**
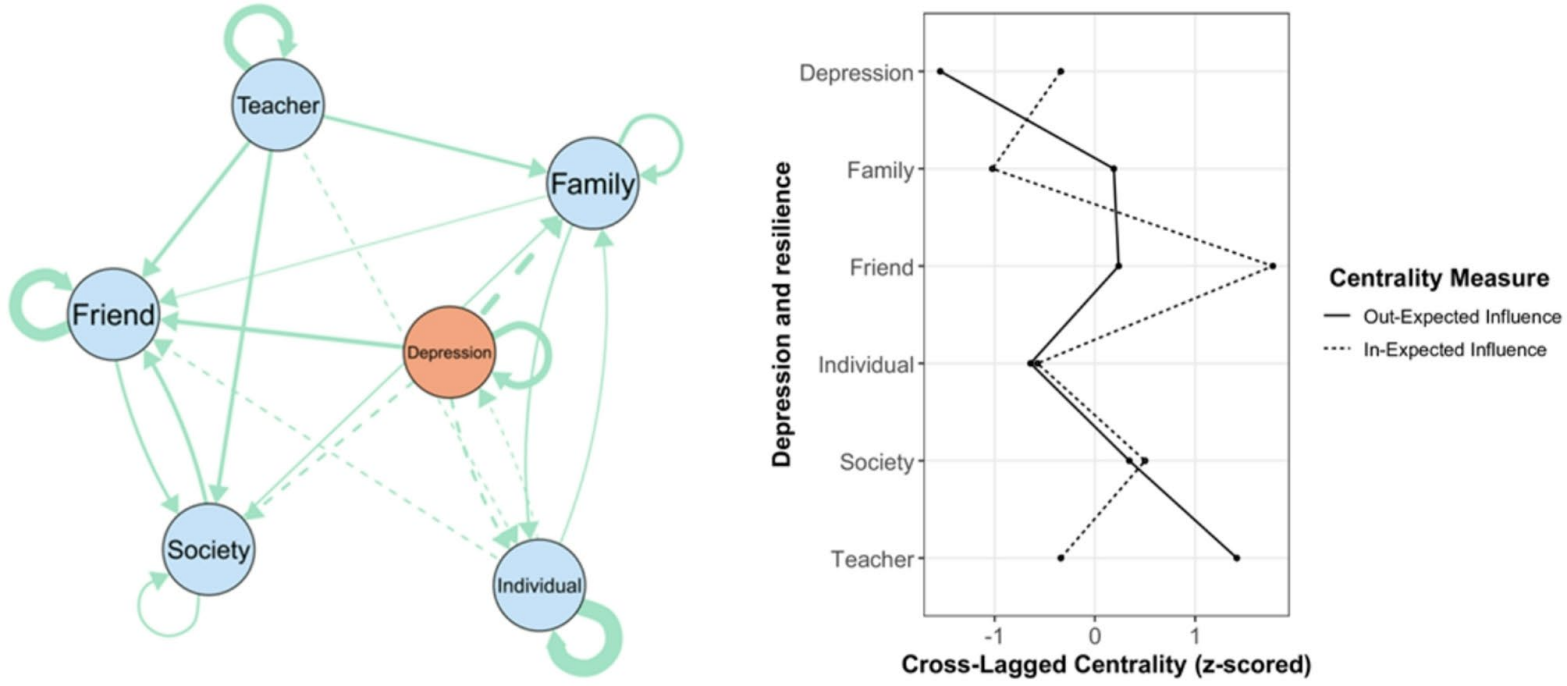
Female sample cross-lag network and Standardized centrality estimates of depressive symptoms network

The male network and standardized centrality estimates are presented in Fig. 2. Friend exhibited the highest out-EI (0.19). Teacher had the highest in-EI (0.27). The two strongest positive associations were Friend → Society (*β* = 0.17), while the two strongest negative associations were Depression → society (*β* = −0.18). In the female network (Fig. 3), teacher exhibited the highest out-EI (0.23) and friend had the highest in-EI (0.35), which was exactly the opposite of typical male network. Regarding edge, the two strongest positive associations were Depression → Friend (*β* = 0.15), whereas the two strongest negative associations were Depression → Family (*β* = −0.19). Across all networks, a negative feedback loop was found to be directly associated with depressive symptoms and individual emotional resilience.

### Stability of three networks

For the overall sample, the CS-coefficients were 0.36 for in-EI and 0.52 for out-EI. Among females, both in-EI and out-EI exhibited high stability, with CS-coefficients of 0.75 for each. Among males, the in-EI stability was 0.44, whereas out-EI remained stable with the value of 0.75.

## Discussion

In this study, we explored the temporal associations between depressive symptoms and multidimensional psychological resilience among adolescents in China, with particular emphasis on gender-specific nuances in the development of the depression-resilience dynamic. Using a longitudinal network approach to study the interplay between depressive symptoms and multi-dimensional psychological factors helps shift from simple preset causal models to recognizing the complex interdependencies among these variables. This shift is significant for promoting personalized and integrated intervention methods. By gaining a more comprehensive understanding of the network structure of depressive symptoms and their psychological factors, more effective intervention strategies can be developed to better meet individual needs [24]. This study demonstrates significant autoregressive effects among various resilience factors, indicating the stability of adolescents’ resilience-related environmental resources. However, the results also reveal significant autoregressive effects among depressive symptoms, which contradicts the findings of previous study [23], this suggests the complexity of adolescent depressive symptom development, which cannot be simply classified as either transient or long-term.

Research has found that teacher and friend support are important resilience factors, which is supported by existing studies [22, 23], further illustrating the importance of establishing a comprehensive support system. In both the overall and female networks, perceived emotional support from teachers had the greatest impact on the overall network, while friendship quality was the most susceptible to interference from other factors. In contrast, among male adolescents, friendship quality was the most intervention-worthy factor. Previous studies had shown that teacher’s support and friendship quality were key connecting hubs in shaping adolescents’ resilience networks [53–56]. A study on teacher-student relationship interventions among female high school students found that improving teacher-student relationships can effectively alleviate depressive symptoms [57].Research has found that male and female have different preferences for support when dealing with environmental resources. Girls are more inclined to seek support that helps them understand and manage their emotions, such as establishing close relationships with authority figures (teachers). Emotional support from teachers can enhance their social confidence, which has increased their positive interaction with environmental factors [58–60]. While boys are more likely to gain emotional support and a sense of belonging through peer relationships. They may expand their social circles and enhances individuals’ sense of social efficacy, establish new social connections, and gain access to more social resources through their friends [55, 56, 61]. Existing research suggests that high-quality friendships may help boys develop stronger psychological resilience compared to girls, possibly due to societal expectations surrounding gender roles [62, 63].

In female networks, a strong predictive relationship was observed between depression and friendship quality. However, among male adolescents, friendship quality showed the strongest predictive effect on social support, forming a positive feedback loop between the two. The co-rumination mechanism is an important pathway for the transmission of depressive symptoms among adolescents. The interpersonal theory [64] of depression suggests that, among adolescents, depression may enhance the closeness of friendships through behaviors such as co-rumination, a phenomenon that is more pronounced among girls [65, 66]. This may be related to the gender role of women being more inclined to express emotions verbally and seek social support [67]. However, it is important to note that while co-rumination may improve friendship quality in the short term, it could exacerbate the risk of depression in the long run [65]. Therefore, attention should be fully focused on the detrimental cycle between girls’ rumination and their friendships during the development of depressive symptoms, and early intervention should be provided. Similarly, training for boys to improve their social skills can enrich and stabilize the quality of their friendships, which in turn can strengthen their social support and enhance environmental resilience systems.

Another finding was that, in female networks, baseline depression levels had a stronger weakening effect on family resilience, whereas among male adolescents, the greatest weakening effect was observed on social support. According to the support erosion model, individuals with depression gradually weaken the support provided by important others due to pessimistic communication, avoidance, or irritability [64, 68]. Previous research has shown that baseline depressive symptoms in adolescents can undermine family functioning, family cohesion, and peer support, but the issue of gender differences has not been explored [69, 70]. In traditional Chinese parenting beliefs, boys are often encouraged to seek independence; therefore, when boys experience depression, they tend to retreat behaviorally and express irritability, which can trigger peer rejection or loss of social opportunities, primarily reflected in the loss of their peer/social support networks. In contrast, girls play the role of “emotional hubs” in family emotional communication, and depressive emotions are more likely to affect family interaction patterns through emotional contagion, leading to an overall deterioration of family functioning [34, 71]. By integrating these sociocultural factors, our findings contribute to a more nuanced understanding of how depression may weaken protective systems in a gender-specific manner within the Chinese context.

In addition to environmental factors, the study also has an interesting finding. In all network groups, a negative feedback loop was identified between depressive symptoms and individual emotional resilience. Depressive symptoms were found to weaken emotional resilience, while reduced emotional resilience, in turn, exacerbated depression. This highlights individual emotional resilience as a highly valuable target for intervention [72].

### Limitations

d be considered when interpreting this study results. Firstly, The research sample was only drawn from a single rural region, and the sample size for subgroups was relatively small. Although the results in this study demonstrated acceptable stability, future research should consider expanding the sample size to different regions for validation. Although we selected key variables such as individual, family, school, peer, and social support based on literature review, some factors were not comprehensively collected, such as individual physiological characteristics and the richness of social support. Additionally, only age and gender were statistically controlled as covariates in the current analysis. Given the multifactorial nature of adolescent depression, future research should adjust for additional relevant variables—such as socioeconomic status, parental education, and baseline mental health status—to better isolate the effects of resilience factors and enhance the robustness of the conclusions. Our research is based on a two-wave data design, but a three-wave study design would provide a more detailed understanding of depressive symptomatology and allow for the examination of the interactions between individual symptoms over time [73]. Additionally, the one-year time interval in this study may limit the generalizability of our findings. Future research should consider a longitudinal design with multiple time intervals within a year (e.g., 3 months, 6 months, 1 year). This approach would help analyze the depression symptoms at multiple time lags, enhancing the robustness of the research findings.

## Conclusion

This study explored the dynamic interactive patterns between teacher support, friend quality, social support, individual emotional resilience, family resilience and depression using cross-lagged network analysis, and also examined gender differences. Research suggests that friendship quality and perceived teacher emotional support are valuable for adolescent resilience networks and depression prevention, but with significant gender differences. Friendship quality is an important intervention target for boys, while teacher emotional support is for girls. Meanwhile, interventions targeting individual emotional resilience can directly and effectively break the vicious cycle between emotional resilience and depressive symptoms.

## Data Availability

The data that support the findings of this study are available from the corresponding author upon reasonable request.
